
JOURNAL INFORMATION
==============================
NLM Title Abbreviation: Exp Physiol
Journal ID: Exp Physiol
NLM Journal ID: 9002940
Journal ID: EPH

Experimental Physiology

ISSN: 0958-0670
EISSN: 1469-445X
Publisher: Wiley

ARTICLE INFORMATION
==============================
PMCID: PMC13327291
DOI: 10.1113/eph.70373
Article ID: EPH70373
Article version: 1
Subjects: Issue Information, Issue Information

Issue Information

Electronic publication date: 2026 Jul 2
Publication date: 2026 Jul
Volume: 111
Issue: 7
First page: 3440
Last page: 3444
Copyright: © 2026 The Authors. Experimental Physiology published by John Wiley & Sons Ltd on behalf of The Physiological Society.
License: This is an open access article under the terms of the http://creativecommons.org/licenses/by/4.0/ License, which permits use, distribution and reproduction in any medium, provided the original work is properly cited.
License URL: https://creativecommons.org/licenses/by/4.0/

Figure count: 0
Table count: 0
Page count: 5

source-schema-version-number: 2.0
cover-date: 1 July 2026
details-of-publishers-convertor: Converter:WILEY_ML3GV2_TO_JATSPMC version:6.8.2 mode:remove_FC converted:02.07.2026

==============================

### Experimental Physiology

Open Access

Volume 111 | Issue 7 | 1 July 2026

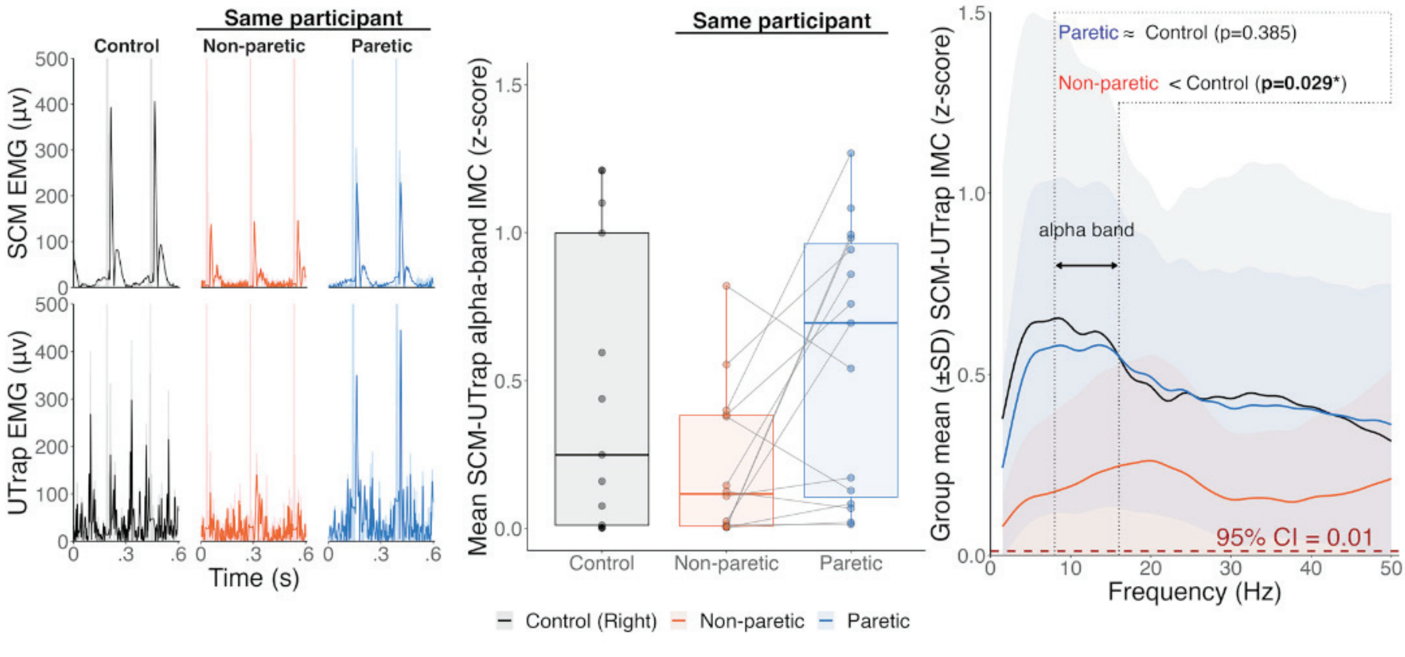

WILEY

The Physiological Society

150 YEARS
1876 2026
LEADING DISCOVERY SCIENCE

### Experimental Physiology

Open Access

Integration and Translation

Volume 111/Issue 7/
1 July 2026

### EDITORIAL BOARD

**EDITOR-IN-CHIEF**
Damian Bailey *Pontypridd, UK*

**DEPUTY EDITOR-IN-CHIEF EUROPE**
Ronan Berg *Copenhagen, Denmark*

**DEPUTY EDITOR-IN-CHIEF USA**
David Poole *Manhattan, KS, USA*

**SENIOR ETHICS EDITOR**
Susan Barman *East Lansing, MI, USA*

**STATISTICAL EDITOR**
Cody Durrer *Copenhagen, Denmark*

**SENIOR REVIEWS EDITOR**
Toby Mündel *Ontario, Canada*

**CONNECTIONS EDITOR**
Prem Kumar *Birmingham, UK*

**SENIOR EDITORS**
Dana Almohazey *Dammam, Saudi Arabia*
Jill Barnes *Madison, WI, USA*
R. Matthew Brothers *Arlington, TX, USA*
Kanokwan 'Mandy' Bunsawat *Iowa City, IA, USA*
Kirstene Callø *Copenhagen, Denmark*
Katrina Colafella *Victoria, Australia*
Ellen Dawson *Liverpool, UK*
Colleen Deane *Southampton, UK*
Peter Zu Eulenburg *Munich, Germany*
Federico Formenti *London, UK*
Julien Ochala *Copenhagen, Denmark*
Shigehiko Ogoh *Kawagoe, Japan*
Jason Peart *Gold Coast, Australia*
Peter Rasmussen *Copenhagen, Denmark*
Lee Romer *London, UK*
Harry B. Rossiter *Los Angeles, CA, USA*
Bill Sheel *Vancouver, Canada*
Mike Stembridge *Cardiff, UK*

**REVIEWING EDITORS**
Tatsuro Amano *Niigata, Japan*
Claire Badenhorst *Palmerston North, NZ*
Tracy Baynard *Boston, MA, USA*
Hannah Caldwell *Copenhagen, Denmark*
Havovi Chichger *Cambridge, UK*
Silvia Conde *Lisbon, Portugal*
Joseph Costello *Portsmouth, UK*
Jennifer Davies *Cardiff, UK*
Judy Delp *Tallahassee, FL, USA*
Aimee Drane *Cardiff, UK*
Esther Dupont-Versteegden *Lexington, KY, USA*
Andrea Fellet *Buenos Aires, Argentina*
Carrie Ferguson *Torrance, CA, USA*
Igor Fernandes *Niteroi, Brazil*
Ryan Hoiland *Kelowna, Canada*
Seth Holwerda *Kansas City, KS, USA*
Anna Hudson *Adelaide, Australia*
Matthew Kay *Washington, DC, USA*
James Lang *Ames, Iowa, USA*
Jacqueline Limberg *Columbia, MO, USA*
Fiona McBryde *Auckland, NZ*
Carmen De Miguel *Birmingham, AL, USA*
Niels Olesen *Copenhagen, Denmark*
Leah Reznikov *Gainesville, FL, USA*
Kleiton Silva *Camden, NJ, USA*
Sarah Short *Iowa City, IA, USA*
Nathaniel Szewczyk *Nottingham, UK*
Can Ozan Tan *Boston, MA, USA*
Mike Tymko *Vancouver, Canada*
Kai Yang *Guangzhou, China*
Song Yao *Melbourne, Australia*
Gensheng Zhang *Hangzhou, China*

**FUTURE LEADERS**
Edward Ashworth *Basel, Switzerland*
Sophie Broome *Melbourne, Australia*
Callum Brownstein *Newcastle, UK*
Daniel Craighead *Minneapolis, MN, USA*
Robert Lakin *Toronto, Canada*
Jedd Pratt *Manchester, UK*
Fabio Sarto *Padua, Italy*
Hollie Speer *Canberra, Australia*

**CONSULTANT EDITORS**
Richard Burden *Bisham, UK*
Nic Green *Bedfordshire, UK*
Julie Greeves *Salisbury, UK*
Denis Noble *Oxford, UK*
Nanduri Prabhakar *Chicago, IL, USA*

**PUBLISHER**
Alex Stewart (astewart@physoc.org)

**DEPUTY MANAGING EDITOR**
Hollie Evans (hevans@physoc.org)

**PEER REVIEW MANAGER**
Diana Jones (ephjournal@physoc.org)

**PEER REVIEW COORDINATOR**
Paroma Guha (ephjournal@physoc.org)

**EDITORIAL OFFICE**
The Physiological Society
Hodgkin Huxley House
30 Farringdon Lane, Clerkenwell
London EC1R 3AW

**PRODUCTION OFFICE**
K. Nandini (EPH@wiley.com)

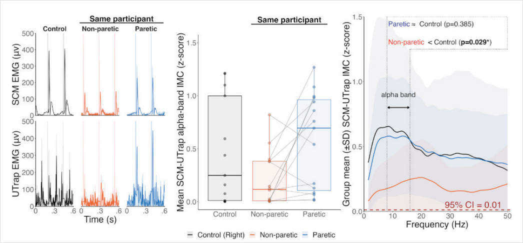

**Cover Illustration**
Galvanic vestibular stimulation reveals disrupted vestibular drive to non-paretic neck muscles following stroke, uncovering disrupted ipsilesional brainstem pathways. See Jiménez et al. Pages 3381–3397.

**Open Access and Copyright**
All articles published by Experimental Physiology [submitted after 18 October 2022] are fully open access: immediately freely available to read, download, and share. All articles [accepted from 18 October 2022] are published under the terms of a Creative Commons license.
Copyright on any research article published by Experimental Physiology is retained by the author(s).
Further information about open access licenses and copyright can be found at: https://authorservices.wiley.com/author-resources/Journal-Authors/licensing/licensing-info-faqs.html#4. Information on the licenses used by Experimental Physiology can be found at: https://physoc.onlinelibrary.wiley.com/journal/1469445X.

For submission instructions and all other information visit: https://physoc.onlinelibrary.wiley.com/journal/1469445X

The Physiological Society and The Physiological Society logo are trade marks belonging to The Physiological Society and are registered in the UK and in the EU Community, respectively.

*Experimental Physiology* publishes high quality, original, physiological research papers that give novel insights into fundamental homeostatic and adaptive responses in health and papers that further our knowledge of pathophysiological mechanisms in diseases.

Experimental Physiology
Open Access
Integration and Translation

### INFORMATION AND GUIDANCE FOR AUTHORS

### Full version online at http://ep.msubmit.net/cgi-bin/main.plex?form_type=display_requirements

### ORIGIN

*The Quarterly Journal of Experimental Physiology*, founded in 1908 by Sir Edward Sharpey-Schafer, Professor of Physiology, University of Edinburgh, became a publication of The Physiological Society in 1981. The title was changed to *Experimental Physiology* in 1990.

### SCOPE

*Experimental Physiology* publishes research papers that report novel insights into homeostatic and adaptive responses in health, as well as those that further our understanding of pathophysiological mechanisms in disease. We encourage papers that embrace the journal's orientation of translation and integration, including studies of the adaptive responses to exercise, acute and chronic environmental stressors, growth and aging, and diseases where integrative homeostatic mechanisms play a key role in the response to and evolution of the disease process. Examples of such diseases include hypertension, heart failure, hypoxic lung disease, endocrine and neurological disorders. We are also keen to publish research that has a translational aspect or clinical application. Comparative physiology work that can be applied to aid the understanding human physiology is also encouraged.

Manuscripts that report the use of bioinformatic, genomic, molecular, proteomic and cellular techniques to provide novel insights into integrative physiological and pathophysiological mechanisms are welcomed.

We are also interested in methodological papers reporting important new developments in physiological techniques that could lead to significant new insights.

Papers which observe a significant new physiological behaviour or phenomenon which has the potential to alter the understanding of that field, but which have not yet identified the mechanisms may also be considered. Such papers should demonstrate the experimental examination and exclusion of a number of plausible mechanisms for the important novel phenomenon.

**Article Types**

In addition to original research, *Experimental Physiology* consider the following article types for publication:

*Case studies, Short Communication, Connections, Reviews, Full Reviews, Hot Topic Reviews Myths and Methodologies and Symposium Reports.*

Review articles are normally by invitation but proposals may be submitted for consideration.

Commentary articles in the form of Viewpoints and Letters are also published.

For full guidelines on all published article see full guidelines.

C O P E COMMITTEE ON PUBLICATION ETHICS

This journal is a member of and subscribes to the principles of the Committee on Publication Ethics

### ETHICS OF EXPERIMENTATION

**Animal Experiments** - see full journal policy at https://physoc.onlinelibrary.wiley.com/hub/animal-experiments

Experimental Physiology endorses the ARRIVE Guidelines for reporting *in vivo* experiments: http://onlinelibrary.wiley.com/doi/10.1113/expphysiol.2010.053793/full

Authors of papers submitted to Experimental Physiology must adhere to the journals' principles and standards for reporting animal experiments.

Authors should read these guidelines to ensure that their research and experiments adhere to our exacting standards.

Euthanasia/humane killing: Animals must be killed using methods approved for that species, stage of development and size. The standards required by the journals are those of the UK/European Union legislation, set out in in ASPA Schedule 1 in the UK and in Annex IV in the European Directive 2010/63/EU. Methods not on Schedule 1 or Annex IV could be permissible but even where the method used conforms with national guidelines, the onus is on the author to provide scientific evidence to demonstrate that it is at least as humane as those on Schedule 1 and Annex IV.

Our guidelines have been outlined in the Editorial 'Principles and standards for reporting animal experiments in Experimental Physiology'.

**Confirmation of compliance**

Authors must state in the Methods section that the investigators understand the ethical principles under which the journal operates and that their work complies with the animal ethics checklist as outlined in the Editorial.

**Human Experiments** - See full journal policy at https://physoc.onlinelibrary.wiley.com/hub/human-experiments

The Editorial Boards are concerned that all work published in The Journal of Physiology and Experimental Physiology should have been conducted according to the highest standards of safety and ethics. Legislation and accepted practice concerning human experimentation vary from country to country and consequently it is difficult to define absolute requirements. However, work with human subjects should conform to the standards set by the Declaration of Helsinki (last modified in 2013), the Medical Research Council's online guidance on the use of human tissue, and the guidelines set out below. The objectives must be to ensure that all risks are minimized and that subjects are not injured and do not feel they have been abused as a result of participating in the study. Any definition of abuse will include excessive or unexpected pain or discomfort experienced during the experiment. Note, in the case of experiments involving minors, any risks must have been considered absent or minimal, and evidence must be presented that the experiments were performed with the understanding and consent of the legal guardian.

All manuscripts must contain statements indicating that (i) informed consent has been obtained, preferably in writing, (ii) studies conformed to the standards set by the Declaration of Helsinki or the version that was in place at the time of the experiments, NOTE: If the research study was registered (clause 35 of the Declaration of Helsinki) the registration database should be indicated, else the lack of registration should be noted as an exception (e.g. The study conformed to the standards set by the Declaration of Helsinki, except for registration in a database.); and (iii) the procedures have been approved by the local ethics committee.

### DISCLAIMER

The Publisher, the Physiological Society and Editors cannot be held responsible for any errors in or any consequences arising from the use of information contained in this journal. The views and opinions expressed do not necessarily reflect those of the Publisher, the Physiological Society or Editors, neither does the publication of advertisements constitute any endorsement by the Publisher, the Physiological Society, Editors, or Authors of the products advertised.

Wiley Open Access articles posted to repositories or websites are without warranty from Wiley of any kind, either express or implied, including, but not limited to, warranties of merchantability, fitness for a particular purpose, or non-infringement. To the fullest extent permitted by law Wiley disclaims all liability for any loss or damage arising out of, or in connection with, the use of or inability to use the content.

Experimental Physiology

Vol 111.7

### Contents of volume 111.7

1 July 2026

### EDITORIAL

3107 From SilverStar to shining stars: Physiology's next generation
*Damian M Bailey*

3111 Recognizing excellence in peer review
*Kim E. Barrett and Damian M. Bailey*

3114 Is there a 'learning effect' on serial lung function assessments?
*Rie Skovly Thomsen, Iben Elmerdahl Rasmussen and Ronan M. G. Berg*

3117 Is altitude-induced polycythaemia an unintended evolutionary mistake?
*Peter D. Wagner and Tatum S. Simonson*

### VIEWPOINT

3122 Development of artificial gravity and resistive vibration exercise protocol: A step towards novel countermeasures for prolonged spaceflight
*Riley A. Dienna, Sabrina K. C. Ehrenfort, Cassandra M. Juran and Scott K. Ferguson*

3125 Can improving your aerobic fitness negate the immediate vascular consequences of prolonged sitting?
*Molly K. Courish and Myles W. O'Brien*

### CONNECTIONS

3127 Recent advances in understanding skeletal muscle ageing: Functional, morphological and omics perspectives
*Oscar Horwath and William Apró*

### MYTHS AND METHODOLOGIES

3130 Myths and methodologies: Exposure and measurements under normobaric hypoxia
*Danilo Bondi*

### METHODS AND TECHNIQUES

3137 The isolated working guinea pig heart: A functional and electrophysiological characterisation
*Grace C. Anderson-Barker and Michael J Shattock*

3152 Development of and adherence to artificial gravity and resistive vibration exercises during 60 days of hypoxic 6° head-down tilt bed rest: BRAVE study
*Jack Fortune, Riccardo G. Sorrentino, Spyridon Zamantzas, Victorien Faivre-Rampant, Lydia Tsoutsoubi, Adam C. McDonnell, Jason T. Fisher, Sara Podgornik, Leonidas G. Ioannou, Urša Ciuha and Igor B. Mekjavic*

### CASE REPORT

3173 Branch retinal artery occlusion due to patent foramen ovale in a teenager
*Jie Zu, Fang Fan, Jiayao Wang, Jianmin Wang and Zhiyang Jia*

### REVIEW ARTICLE

3179 Neuroprotective roles of klotho: Molecular pathways and therapeutic implications for cognitive health in neurological and psychiatric diseases
*Amir Arsalan Ghahari, Leylinaz Safaeihavadaragh, Mehrdad Nourizadeh, Shaghayegh Davari, Saba Safaei, Farshad Zare, Safa Alkayyat and Erfan Kamali Far*

3191 Blue plaque review series: Thomas Graham Brown: Before his time
*Ronald L. Calabrese and Eve Marder*

### RESEARCH ARTICLE

### CARDIOVASCULAR CONTROL

3203 Higher body-mass index modifies time-resolved maternal autonomic cardiac–uterine coupling during the first stage of human labour
*Carlos Gabriel Varela-Albarrán, José Javier Reyes-Lagos, Laura Mercedes Santiago-Fuentes, Guadalupe Dorantes-Méndez, Eric Alonso Abarca-Castro, Paula Romina Soria and Araceli Espinosa-Guerrero*

3216 Oxidative stress and impaired cardiovagal baroreflex sensitivity in young adults with post-traumatic stress disorder
*Jennifer B. Weggen, Jacob W. Richardson, Emily A. Buck, Brad T. Bakken, Brandon J. Fitzpatrick, Raven G. Campbell and Ryan S. Garten*

### ENVIRONMENTAL AND EXERCISE

3228 Influence of oestradiol on exercise induced muscular damage and heart rate variability in a non-trained healthy population
*David Ramiro-Cortijo, Ricardo Alonso de Celada, Pilar Rodríguez-Rodríguez, Stefanny Lozano Gutiérrez, Leonardo Betancourt, Santiago Ruvira and Silvia M. Arribas*

3242 Eight weeks of high-intensity interval training is insufficient to prevent sitting-induced endothelial dysfunction and microvascular impairment
*Nobukazu Kasai, Hayato Ihara, Motoyuki Iemitsu and Takuma Morishima*

3253 $\dot{V}_{O_{2max}}$-based all-out aerobic exercise attenuates hexosamine biosynthetic pathway activity: Metabolomic insights in mice model
*Gyumin Kang, Eui-Cheol Shin, Jae Kyeom Kim and Young Jun Kim*

3267 Cerebral oxygenation and skeletal muscle responses to time trial, maximal oxygen uptake and time to exhaustion exercise
*Caroline V. Robertson, Nicole T. Vargas, Tegan Hartmann and Frank E. Marino*

3280 Advancing tele-physiology: A chest patch solution for continuous, non-invasive remote monitoring in a hypoxic environment
*Danilo Bondi, Ferdinando Calcagno, Elisa Fioraso, Camillo Di Giulio, Federico Schena and Anna Pedrinolla*

### HEART/CARDIAC MUSCLE

3294 Glycosylation of the murine cardiac channel TRPM4 is altered by the pathogenic p.I376T variant
*Sabrina Guichard, Emanuele di Lorenzo, Dominic Schneiter, Maria Essers, Prakash Arullampalam, Jean-Sébastien Rougier and Hugues Abriel*

3310 Regeneration of the lizard heart after cryoinjury
*Martina Gregorovicova, Barbora Sankova, Martin Bartos, Bjarke Jensen, Tobias Wang and David Sedmera*

### MUSCLE

3322 Ischaemic pre-conditioning improves workload, fatigue and recovery in team sport: A randomized, double-blind, placebo-controlled crossover trial
*Lorcan Daly, Shane Farrell and Alexander Gamble*

3337 Effects of chemical myosin II cross-bridge inhibitors on eccentric force production in skinned muscle fibres
*André Tomalka, Sven Weidner, Christian Rode and Tobias Siebert*

3357 Skeletal muscle-specific myostatin overexpression promotes muscle oxidative capacity and fatigue resistance in transgenic mice
*Andy V. Khamoui, Andrea Abraham, Janos Porszasz, Istvan Kovanecz, Silvana Constantinescu, Harry B. Rossiter and Suzanne Reisz-Porszasz*

### NEUROSCIENCE

3381 Galvanic vestibular stimulation reveals disruption of ipsilesional brainstem pathways in hemiparetic stroke survivors
*Angelo Bartsch-Jiménez, Hesam Azadjou and Francisco J. Valero-Cuevas*

### RENAL

3398 Impact of recurrent dehydration with mild periodic water restriction on blood pressure and renal function in male spontaneously hypertensive rats
*Katrina M. Mirabito Colafella, Peter N. H. Nguyen, Zoe McArdle, Debra Fong, Edmund Kwok, Kate M. Denton, Antony Vinh and Lucinda M. Hilliard Krause*

### RESPIRATORY

3412 The effect of pre-exercise hyperventilation on repeated high-intensity inclined sprint performance
*Leon Fesseler, Andreas Patzak, Viktor Heinz, Ngan-Ha Stella Le, Oliver Opatz, Niklas Pilz and Tomas Lucca Bothe*

### LETTER TO THE EDITOR

3425 Interpreting whole-body carbohydrate oxidation and ‘oxidation efficiency’ in carbohydrate supplement studies
*Stefan Pettersson*

3427 Prespecified analysis plans are not suitable for exploratory experimental physiology research
*Erik A. Richter, Bente Kiens and Abigail L. Mackey*

### REPLY

3429 Authors reply to: Interpreting whole-body carbohydrate oxidation and ‘oxidation efficiency’ in carbohydrate supplement studies
*Ewan Dean, Ash Osbrone, Daren Subar, Paul Hendrickse and Christopher J. Gaffney*

3431 Transparency as the foundation of discovery
*Peter Rasmussen, Damian Miles Bailey and Alex Stewart*

3434 Why reporting requires more than findings
*Robin Christensen, Tobias Haugegaard and Janus C. Jakobsen*

3437 Reply to letter to the editor in response to: “Carbon monoxide to ‘simulate altitude’”
*Simone Villanova, Simone Porcelli, Lena Ekström and Daniele A. Cardinale*

### CORRIGENDUM

3439 Correction to ‘Resting metabolic rate prediction equations and the validity to assess energy deficiency in the athlete population’

Editor’s Pick

Galvanic vestibular stimulation reveals disrupted vestibular drive to non-paretic neck muscles following stroke, uncovering disrupted ipsilesional brainstem pathways. See Jiménez et al. Pages 3381–3397.
